# Identification and Expression Profiling of the BTB Domain-Containing Protein Gene Family in the Silkworm, *Bombyx mori*

**DOI:** 10.1155/2014/865065

**Published:** 2014-05-06

**Authors:** Daojun Cheng, Wenliang Qian, Meng Meng, Yonghu Wang, Jian Peng, Qingyou Xia

**Affiliations:** State Key Laboratory of Silkworm Genome Biology, Southwest University, No. 2 Tiansheng Street, Beibei District, Chongqing 400715, China

## Abstract

The BTB domain is a conserved protein-protein interaction motif. In this study, we identified 56 BTB domain-containing protein genes in the silkworm, in addition to 46 in the honey bee, 55 in the red flour beetle, and 53 in the monarch butterfly. Silkworm BTB protein genes were classified into nine subfamilies according to their domain architecture, and most of them could be mapped on the different chromosomes. Phylogenetic analysis suggests that silkworm BTB protein genes may have undergone a duplication event in three subfamilies: BTB-BACK-Kelch, BTB-BACK-PHR, and BTB-FLYWCH. Comparative analysis demonstrated that the orthologs of each of 13 BTB protein genes present a rigorous orthologous relationship in the silkworm and other surveyed insects, indicating conserved functions of these genes during insect evolution. Furthermore, several silkworm BTB protein genes exhibited sex-specific expression in larval tissues or at different stages during metamorphosis. These findings not only contribute to a better understanding of the evolution of insect BTB protein gene families but also provide a basis for further investigation of the functions of BTB protein genes in the silkworm.

## 1. Introduction

The BTB (bric-a-brac, tramtrack, broad complex) domain, also known as the POZ domain, is an evolutionarily conserved protein-protein interaction motif consisting of approximately 100 amino acid residues. This domain was originally identified in the bric-a-brac, tramtrack, and broad complex proteins in the fruit fly (*Drosophila melanogaster*) [1]. The BTB domain-containing protein (referred to as “BTB protein” hereafter) gene family is characterized by the presence of one or more BTB domains in each family member and is found widely in eukaryotes [1–6]. Several other types of functional domains, including zinc finger (ZF), Kelch, BTB and C-terminal Kelch (BACK), meprin and TRAF homology (MATH), ankyrin repeats (ANK), PHR, and Ras homology (Rho) domains, are also found in some BTB proteins. According to the presence of these domains, the BTB protein gene family can be divided into multiple subfamilies, including BTB only proteins, BTB-ZF proteins, BTB-Kelch proteins, BTB-BACK proteins, BTB-BACK-Kelch proteins, MATH-BTB proteins, BTB-ANK proteins, BTB-BACK-PHR proteins, and Rho-BTB proteins [3, 5, 6].

The members of the BTB protein gene family are functionally involved in a variety of biological events in eukaryotes, including developmental timing [7], oogenesis [8], organ formation [9], cell development [10, 11], cell apoptosis [12, 13], protein degradation [14–17], cytokinesis [18, 19], and tumorigenesis [20, 21]. Furthermore, the functions of BTB proteins are mainly governed by two prominent mechanisms [5]: BTB domain-based protein-protein interactions [22–24] and transcriptional regulation by DNA binding domains such as zinc finger domains [6, 25–28].

Genome-wide characterization of the BTB protein gene family has been performed in several species. The numbers of genes belonging to this family vary greatly among species [6]; 183 are found in humans (*Homo sapiens*), 178 in the nematode (*Caenorhabditis elegans*), 195 in the mouse (*Mus musculus*), 77 in the mouse-ear cress (*Arabidopsis thaliana*), 85 in the fruit fly, and 85 in the malaria mosquito (*Anopheles gambiae*). The silkworm (*Bombyx mori*) is considered an economically important insect and has been used widely for determining the genetic basis of biological events in the order Lepidoptera. To date, only one BTB protein, broad complex (BR-C) protein of the BTB-ZF subfamily, has been identified in the silkworm, where it has been shown to mediate ecdysone signaling [29, 30]. In the present study, we systematically identified BTB protein genes in the silkworm using the silkworm genome sequence [31], compared them with the BTB protein genes of other insects, and profiled their spatial and temporal expression patterns.

## 2. Materials and Methods

### 2.1. Genome-Wide Identification of BTB Protein Genes

BTB protein genes were identified in thesilkworm genome using the amino acid sequence of the conserved BTB domain as a query against the gene collections downloaded from two silkworm genome databases, SilkDB (http://www.silkdb.org/silkdb/) and KAIKObase (http://sgp.dna.affrc.go.jp/KAIKObase/), in a local BLASTp search. The predicted BTB protein genes were further annotated through an online BLAST search in NCBI. The same approach was used to identify BTB protein genes in four other insects: the fruit fly, the honey bee (*Apis mellifera*, Hymenoptera), the red flour beetle (*Tribolium castaneum*, Coleoptera), and the monarch butterfly (*Danaus plexippus*, Lepidoptera). Two online programs, SMART (http://smart.embl-heidelberg.de/) [32] and a CDD search program (http://www.ncbi.nlm.nih.gov/Structure/cdd/wrpsb.cgi) [33], were used to characterize the domain architecture of the predicted BTB protein genes in the silkworm genome. According to the gene-naming principles established by the Gene Nomenclature Committee of the Human Genome Organization (HGNC), “BTB” plus the initial word in the descriptions of other domains was used for the abbreviated names of the BTB protein genes identified in this study.

### 2.2. Chromosomal Distribution and Subcellular Localization

Silkworm BTB protein genes were mapped on the chromosomes based on the single- nucleotide polymorphism (SNP) linkage map for the silkworm [34]. The subcellular locations and other characteristics of silkworm BTB proteins were predicted by two programs: WoLF PSORT (http://wolfpsort.org) [35] and TargetP (http://www.cbs.dtu.dk/services/TargetP).

### 2.3. Construction of Phylogenetic Trees

The full amino acid sequences and BTB domain sequences of the identified BTB proteins of the silkworm and other surveyed insects were used to perform a multiple sequence alignment by the ClustalX program [36]. The alignment result was further used to build a neighbor-joining phylogenetic tree with a bootstrap of 1,000 replicates. The phylogenetic tree was refined by the MEGA4 program [37].

### 2.4. Microarray Analysis of Spatiotemporal Gene Expression

Based on microarray data for gene expression in various tissues of silkwormlarvae on day 3 of the fifth instar (L5D3) [38], we determined the expression patterns of silkworm BTB protein genes on L5D3 in nine larval tissues, including ovary, testis, head, integument, fat body, midgut, hemocyte, Malpighian tubule, anterior/middle silk gland (A/MSG), and posterior silk gland (PSG). The approach used in this analysis was identical to that described in a previous report [38]. In addition, based on the microarray data for gene expression during silkworm metamorphosis, which involves the transition from late larva to pupa and then to adult (unpublished), we analyzed the expression patterns of silkworm BTB protein genes at 19 developmental time points. These time points included day 4 of the fifth larval instar: (L5D4), L5D5, L5D6, L5D7, beginning of wandering for spinning (W0), 12 hours after wandering (W12 h), W24 h, W36 h, W48 h (completing spinning), beginning of pupation (P0), day 1 following pupation (P1), P2, P3, P4, P5, P6, P7, P8, and adult. Gene expression in the silkworm individuals on L5D3 was used as a control. Microarray hybridization and raw data processing were performed as previously described [38]. If the signal intensity of the expression of a BTB protein gene exceeded 400 units at a time point, this gene was considered to be expressed at that time point. We then calculated the ratio of the expression level of each BTB protein gene inthe silkworm individuals at each time point to that in the L5D3 control. These ratios were further used to examine dynamic changes in the expression levels of BTB protein genes during silkworm metamorphosis using the GeneCluster 2.0 program [39].

### 2.5. RT-PCR Examination of Spatiotemporal Gene Expression

RT-PCR experiments were further used to examine the expression patterns of silkworm BTB protein genes in multiple tissues of silkworm larvae on L5D3. Silkworms of the* Dazao* strain were reared on fresh mulberry leaves at 25°C under 12-hour light/12-hour dark cycle. Silkworm larval tissues, including silk gland, midgut, fat body, hemocyte, head, integument, Malpighian tubule, ovary, and testis, were isolated from female and male larval individuals on L5D3. The procedures for RNA extraction, cDNA synthesis, and RT-PCR reaction were the same as previously described [40]. The primers used in these experiments are listed in Table S1 (see Table S1 in Supplementary Material available online at http://dx.doi.org/10.1155/2014/865065).

## 3. Results

### 3.1. Identification of BTB Protein Genes in the Silkworm Genome

**Figure 1:**
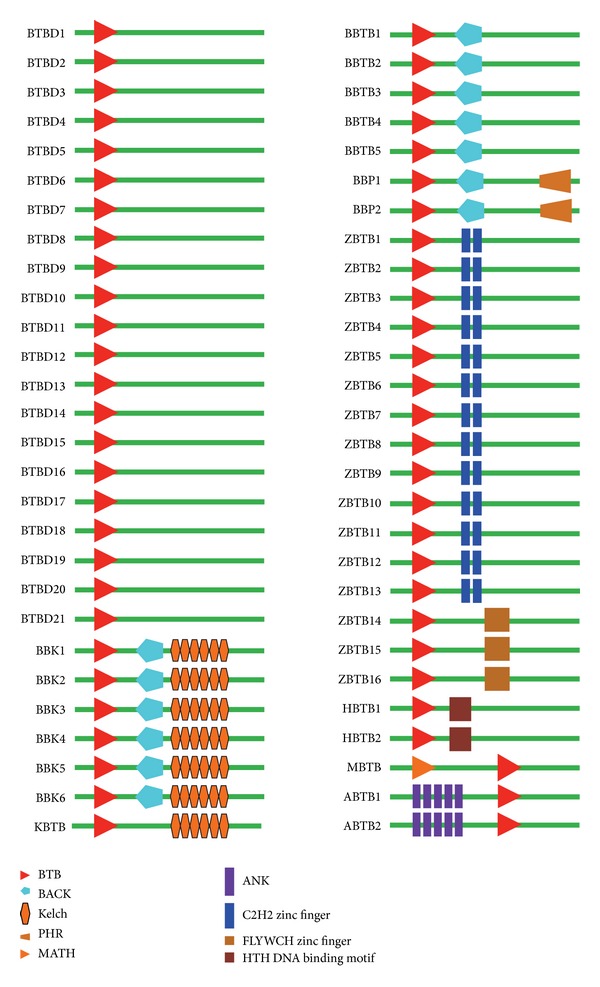
Architecture of functional domains in silkworm BTB protein genes. The conserved BTB domain and other types of functional domains are marked with different signs and colors as indicated below the schematic architecture. Domain names refer to the resource from the SMART database.

To identify BTB protein genes in the silkworm genome, we used the amino acid sequence of a common BTB domain (SMART accession number: SM00225) and the BTB domain sequence of BR-C protein as queries to search against the predicted silkworm gene collection. As a result, 56 BTB protein genes were retrieved from the silkworm genome (Table 1). SMART and CDD search-based domain analysis showed that, in addition to the BTB domain, most silkworm BTB protein genes also contained one or more other functional domain types such as ANK, BACK, PHR, Kelch, zinc finger, HTH DNA binding motif, and MATH domains (Table 1 and Figure 1).

According to previous reports [6] and on the basis of the presence of other types of domain, we classified the identified silkworm BTB protein genes into nine subfamilies: BTB only (BTBD), BTB-ANK (ABTB), BTB-BACK (BBTB), BTB-BACK-PHR (BBP), BTB-BACK-Kelch (BBK), BTB-Kelch (KBTB), BTB-ZF (ZBTB including those with C2H2 or FLYWCH zinc finger domains), BTB-HTH (HBTB), and MATH-BTB (MBTB) subfamilies. The silkworm BTB protein gene subfamilies were named in an abbreviated format based on the principles for the nomenclature of BTB proteins described by the HUGO Gene Nomenclature Committee (HGNC) and on previous reports [6]. These nine subfamilies contain 22, 2, 5, 2, 6, 1, 15, 2, and 1 members, respectively (Table 1 and Figure 1).

### 3.2. Chromosomal Distribution and Subcellular Location of Silkworm BTB Protein Genes

**Figure 2:**
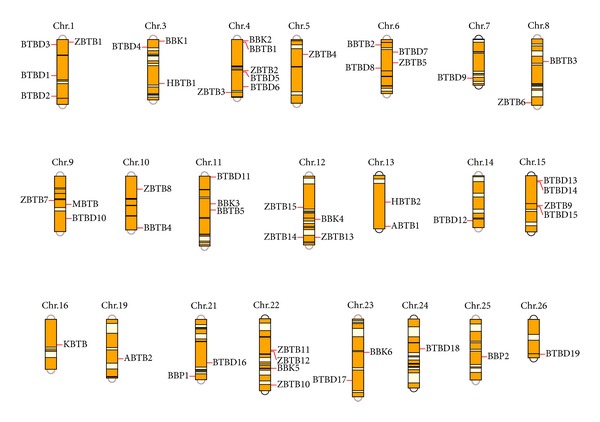
Chromosomal distribution of silkworm BTB protein genes. In total, 53 of the identified silkworm BTB protein genes (i.e., all except* BTBD20*,* BTBD21*, and* ZBTB13*) were localized to specific chromosomes based on the whole-genome sequence and single-nucleotide polymorphism (SNP) marker linkage map for the silkworm. The chromosome number of each gene was indicated above.

Based on the linkage map [34] and the updated genome assembly for the silkworm [31], 53 of the 56 identified silkworm BTB protein genes (excluding* BTBD20*,* BTBD21*, and* ZBTB13*) were assigned to 22 chromosomes (Table 1 and Figure 2). The members of each BTB protein subfamily were distributed dispersedly on the different chromosomes.

We predicted the subcellular location of silkworm BTB proteins using the online programs WoLF PROST and TargetP. As expected, BTB proteins containing C2H2 zinc finger or HTH DNA binding motifs, two domains associated with activation of the transcription of target genes, were predicted to be located within the nucleus (Table 1); the BTB-ANK and MATH-BTB subfamilies were also classified among the nuclear proteins. However, BTB proteins containing the FLYWCH zinc finger domain and the members of the BTB-BACK-PHR subfamily were predicted to be located in the cytosol. The members of each of the remaining BTB protein subfamilies were assigned to the nucleus, cytosol, or mitochondrion.

### 3.3. Phylogenetic Tree of Silkworm BTB Protein Genes

**Figure 3:**
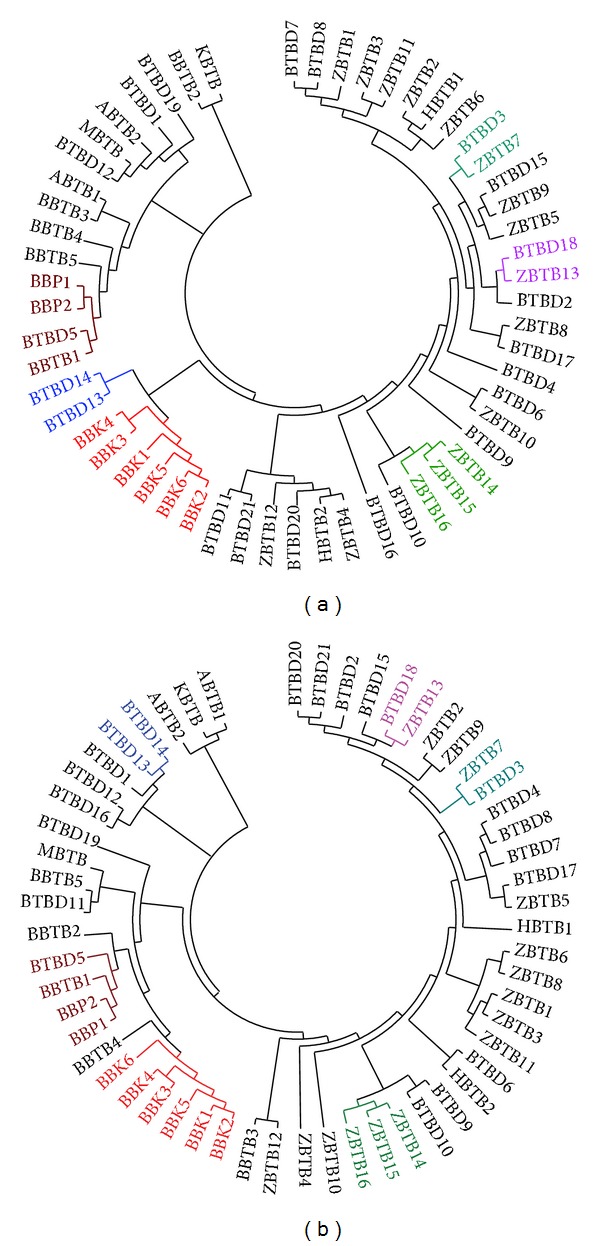
Phylogenetic tree of silkworm BTB protein genes. A neighbor-joining phylogenetic tree of the identified silkworm BTB protein genes was constructed using the ClustalX program and visualized using the MEGA4 software. (a) BTB domain sequence-based phylogenetic tree. (b) Complete amino acid sequence-based phylogenetic tree. Similar grouping clades between these two phylogenetic trees are indicated by the same colors.

Given that BTB fold of the BTB proteins has been shown to be structurally well conserved [6], we used the amino acid sequences of BTB domains from the identified silkworm BTB proteins to generate a phylogenetic tree of silkworm BTB protein genes. As shown in Figure 3(a), the members of several BTB protein gene subfamilies, including BTB-BACK-Kelch, BTB-BACK-PHR, and BTB-FLYWCH proteins (*ZBTB14*,* ZBTB14*, and* ZBTB15*) of the BTB zinc finger subfamily, were grouped more closely than other BTB protein genes. Curiously, the members of each of the remaining subfamilies, except for BTB-Kelch and MATH-BTB, each of which contained single gene, were grouped irregularly with the members of other subfamilies.

We also constructed a phylogenetic tree of silkworm BTB protein genes using the complete amino acid sequences predicted by these genes. As shown in Figure 3(b), the members of three subfamilies, BTB-BACK-Kelch, BTB-BACK-PHR, and BTB-FLYWCH, were tightly grouped together, which is consistent with the observation from the BTB domain sequence-based phylogenetic tree. This indicates that the BTB protein genes in these three subfamilies have experienced a duplication event and may have similar biological functions. Moreover, several grouping clades were also similar to those indicated by the BTB domain-based phylogenetic tree, including the clade of* BTBD13* and* BTBD14*, the clade of* BTBD3* and* ZBTB3*, the clade of* BTBD18* and* ZBTB13*, and the clade including* BTBD5*,* BBTB1*, and the BTB-BACK-PHR subfamily. The remaining BTB protein genes were grouped into different clades in an irregular manner.

### 3.4. Comparison of the BTB Protein Genes of the Silkworm with Those of Other Insects

To comprehensively characterize the evolution of insect BTB protein genes, we identified BTB protein gene families in four insect species for which the whole-genome sequence is available. As listed in Table 2 and Table S2, 44 BTB protein genes were found in the fruit fly, 46 in the honey bee, 55 in the red flour beetle, and 53 in the monarch butterfly. Intriguingly, the BTB-ANK and MATH-BTB subfamilies contained the same numbers of genes, two and one, respectively, in the silkworm and in the other four surveyed insect species. Furthermore, the BTB-Kelch subfamily was found in only three of the four additional surveyed insect species (not in the honey bee). The BTB-BACK-PHR subfamily was found only in the silkworm, honey bee, and monarch butterfly, whereas the RhoBTB subfamily was present in the fruit fly, red flour beetle, and monarch butterfly.

**Figure 4:**
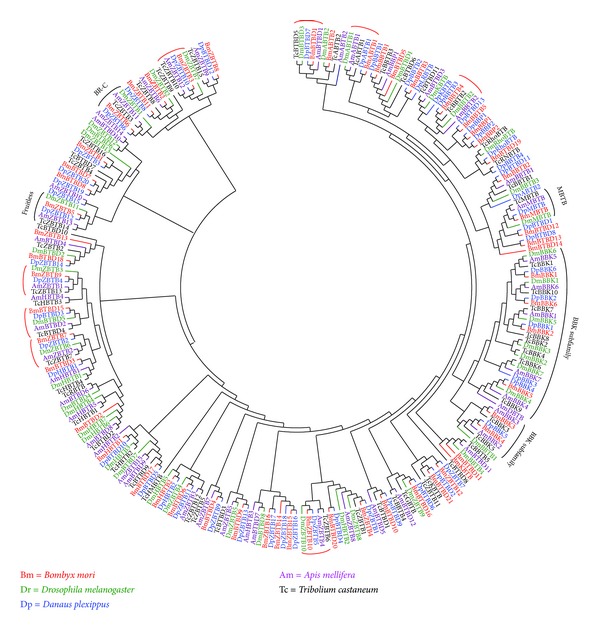
Phylogenetic relationships of the BTB protein genes of the silkworm with those of other surveyed insects. Based on the amino acid sequences of the BTB domains, a neighbor-joining phylogenetic tree of all insect BTB protein genes was constructed using the ClustalX program and visualized using the MEGA4 software. The grouping clade for the orthologs of a silkworm BTB protein gene that showed a 1 : 1 : 1 : 1 : 1 orthologous relationship among all five surveyed insect species is indicated by a red cambered line. The names of all BTB protein genes from a single insect species are indicated in the same color.

We constructed a phylogenetic tree including all of the BTB protein genes identified in the silkworm and other surveyed insects using the amino acid sequences of their BTB domains (Figure 4). In this phylogenetic tree, the members of two subfamilies, MATH-BTB and BTB-BACK-Kelch, were always grouped together. Furthermore, although most members of the BTB-ZF subfamily were promiscuously grouped into different clades with members of other BTB subfamilies,* BmZBTB6* and* BmZBTB5*, two silkworm BTB-ZF protein genes that encode BR-C and fruitless, respectively, were grouped together into a clade with their homologs from other surveyed insects and they showed an orthologous 1 : 1 : 1 : 1 : 1 relationship among all five insect species. This was the same as grouping clades containing the orthologs of the following BTB protein genes from the silkworm:* BmZBTB3*,* BmZBTB7*,* BmZBTB9*,* BmZBTB10*,* BmBTBD1*,* BmBTBD15*,* BmABTB1*, and* BmBBTB4*.

### 3.5. Tissue Expression Profile of Silkworm BTB Protein Genes

**Figure 5:**
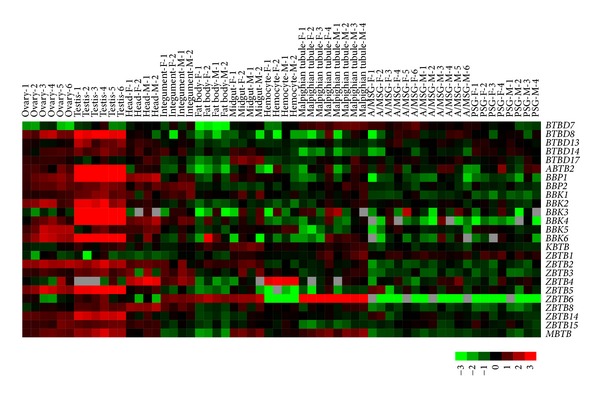
Microarray-based expression profiling of BTB protein genes in multiple tissues of silkworm larvae. Microarray data representing genome-wide gene expression in multiple tissues of silkworm larvae on day 3 of the fifth instar were downloaded from the SilkDB database. Each tissue sample was analyzed using at least two biological repeats, which are indicated with different Arabic numerals. F: female; M: male; A/MSG: anterior/median silk gland; PSG: posterior silk gland.

**Figure 6:**
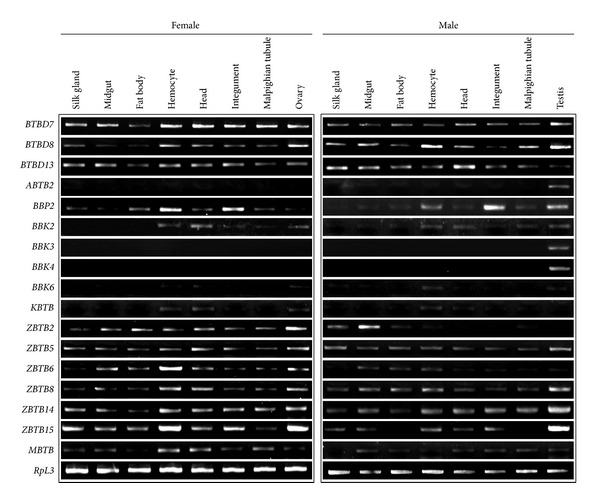
RT-PCR examination of expression profiles of the BTB protein genes in silkworm larval tissues. The expression profiles of the BTB protein genes in multiple tissues of silkworm larvae on day 3 of the fifth instar (L5D3) were further examined in RT-PCR experiments. The silkworm* RpL3* gene was used as a control.

A previous study reported a microarray analysis of genome-wide gene expression in multiple tissues of silkworm larvae on day 3 of the fifth instar (L5D3) [38]. Based on microarray data from that study, we analyzed the expression patterns of silkworm BTB protein genes in larval tissues and found that 25 of the identified silkworm BTB protein genes were expressed in at least one larval tissue (Figure 5). Notably, 22 BTB protein genes were highly expressed in the gonads of the silkworm, and most of these genes showed higher expression in the testis than in the ovary. Interestingly,* ABTB2*,* BBK3*, and* BBK4* were specifically expressed in the testis.* ZBTB6* expression was present in the Malpighian tubule, integument, fat body, and midgut.* ZBTB4* was mainly expressed in the hemocytes, head, and integument.* MBTB* showed ubiquitous expression in the ovary, testis, midgut, and Malpighian tubule. In addition, RT-PCR examination confirmed the testis-specific expression of* ABTB2*,* BBK3*, and* BBK4* in silkworm larvae (Figure 6).

### 3.6. Developmental Expression Profile of the Silkworm BTB Protein Genes

**Figure 7:**
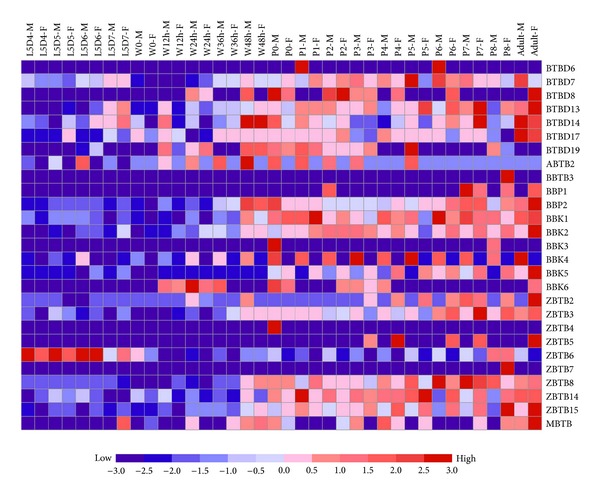
Microarray-based expression profiling of the BTB protein genes during silkworm metamorphosis. Time-course microarray data representing gene expression during silkwormmetamorphosis was used to profile the developmental expression patterns of silkworm BTB protein genes. Changes in the expression of the BTB protein genes were visualized using the GeneCluster 2.0 program. Plus and minus denote up- and downregulation, respectively. F: female; M: male; L: larval; W: wandering; P: pupal; D: day; h: hour. The Arabic numeral in phase P1 represents one day after pupation.

Using the microarray data on gene expression during silkworm metamorphosis from the larval to adult stages, we surveyed the developmental expression profile of the silkworm BTB protein genes. The results showed that 27 of the identified BTB protein genes were expressed during silkworm metamorphosis (Figure 7). Remarkably, most of these BTB protein genes showed a high expression during the pupa-adult transition. Moreover, the expression of eight silkworm BTB protein genes, including* BTBD6*,* ABTB2*,* BBTB3*,* BBK3*,* BBK4*,* BBK5*,* ZBTB5*, and* ZBTB7*, displayed sexual dimorphism; the expression of* BTBD6*,* ABTB2*,* BBK3*, and* BBK4* was male-specific, whereas that of* BBTB3*,* BBK5*,* ZBTB5*, and* ZBTB7* was female-specific.

## 4. Discussion

Members of the BTB protein gene family are characterized by the presence of a BTB domain that mediates protein-protein interactions [22, 41, 42]. In this study, 56 BTB protein genes were identified in the silkworm genome. Similar to the findings of a previous study [6], in addition to the typical BTB domain, most of the identified silkworm BTB protein genes also contained other types of functional domains such as zinc finger, BACK, Kelch, ANK, MATH, PHR, and HTH domains. Based on the presence of the other domains, we classified the identified silkworm BTB protein genes into nine subfamilies: BTB only, BTB-ANK, BTB-BACK, BTB-BACK-PHR, BTB-BACK-Kelch, BTB-Kelch, BTB-ZF, BTB-HTH, and MATH-BTB. The presence of various additional functional domains in the silkworm BTB protein genes suggests that the BTB protein genes in different subfamilies may have diverse functions.

Our comparative analysis revealed that BTB protein genes have been conserved to a certain extent during insect evolution. This conclusion is supported by the following evidence. First, the total number of BTB protein genes present in the silkworm genome and in the genomes of four other surveyed insect species is similar, indicating that no expansion has occurred in the BTB protein gene family during insect evolution. This finding differs from a previous observation that BTB protein genes have undergone a lineage-specific expansion in vertebrates in a comparison with the fruit fly and the malaria mosquito [6]. Second, we noted that the members of some BTB protein gene subfamilies showed a rigorous orthologous relationship among the silkworm and other insects, suggesting that these members may have similar functions in the surveyed insect species. For example, the orthologs of* BR-C* (*ZBTB6*) or* fruitless* (*ZBTB5*), two well-studied insect BTB protein genes, were grouped into a clade on the phylogenetic tree (Figure 4). This finding is consistent with the known conserved functions of BR-C protein in ecdysone signaling [7] and with the contribution of fruitless protein to the regulation of male sexual behavior and sexual orientation [43]. In addition, previous studies have shown that MATH-BTB proteins are ubiquitously present in plants and metazoa [6] and they mainly function as substrate-binding adapters for the Cullin3-based ubiquitin E3 ligase that is involved in the ubiquitination and proteolysis of target proteins [44–46]. Because ubiquitination-induced protein degradation also occurs in the fruit fly [47, 48], we reasoned that MATH-BTB protein-mediated ubiquitination should occur widely in insects. Third, in all insect species examined, the BTB-BACK-Kelch subfamily possesses a similar number of members and has experienced a moderate expansion when comparing to this subfamily in yeast [6]. This observation, coupled with the finding that the BTB-BACK-Kelch subfamilies in the surveyed insects were grouped together in the phylogenetic tree of all insect BTB protein genes but form different subclades based on sequence similarity and not species identity (Figures 3 and 4), suggests that the expansion of the BTB-BACK-Kelch subfamily has occurred before insect radiation.

Our work also shows that evolutionary divergence has also occurred among insect BTB protein genes. First, in agreement with previous observations [6], the number of BTB protein genes in the silkworm and other insect species is apparently smaller than that in mammals and larger than that in yeast, further confirming the occurrence of lineage-specific expansions of BTB protein genes. Second, different members of the BTB only subfamily were always separately grouped with the members of other subfamilies in the phylogenetic trees constructed either from silkworm BTB protein genes or from all insect BTB protein genes, suggesting that the BTB protein genes that contain additional domains may have evolved from these BTB protein genes of the BTB only subfamily. Similarly, some members of the BTB-ZF subfamily were also grouped together with the members of BTB only proteins before grouping with other members of BTB-ZF subfamily in the phylogenetic tree based on the complete amino acid sequences of silkworm BTB proteins, indicating that these BTB-ZF proteins may play distinct roles. Third, we noted that the BTB-BACK-PHR and RhoBTB subfamilies were not present in some insect species. Whether this lack resulted from the genome gap or whether these subfamilies actually play specific roles in the insect species in which they are present remains to be determined.

Another important finding of our study is that several silkworm BTB protein genes exhibited spatiotemporally specific expression profiles. Interestingly, silkworm* ABTB2*,* BBK3*, and* BBK4* were expressed specifically in the testis of mid-fifth instar larvae and in male individuals during metamorphosis. The silkworm* ABTB2 *gene encodes an inhibitor of Bruton tyrosine kinase (BTK), which has been shown to be involved in male genital development and survival in the fruit fly [49, 50]. Therefore, the male-specific expression of the silkworm* ABTB2* gene suggests that this gene may be involved in the regulation of male genital development in the silkworm. In addition, we noted that* ZBTB5 *gene showed no sex-dependent expression on L5D3 but was specifically expressed in the female silkworm during the pupa-adult transition. Given that during oogenesis the growth and meiosis of the oocytes and egg formation occur during the pupa-adult transition of the silkworm [51], we speculate that* ZBTB5 *may play key roles in the regulation of silkworm oogenesis. Future studies are necessary to determine how sex-specific expression of these BTB protein genes functions during silkworm development.

## Supplementary Material

Table S1: List of the primers used in this study.
Table S2: Inventory of BTB protein genes in other four insect species, including the fruit fly, honey bee, red flour beetle, and monarch butterfly.

## Figures and Tables

**Table 1:** Inventory of BTB protein genes in the silkworm genome.

Subfamily	Proposed name	SilkDB ID	KAIKObase ID	Amino acid residues (aa)	Molecular weight (Da)	PI	Subcellular localization	Domain	Chromosome
BTB only	BTBD1	BGIBMGA000517	Gene000396	1812	200095	6.01	nucl	BTB	1
	BTBD2	BGIBMGA012231	Gene000660	351	38870	10.06	mito	BTB	1
	BTBD3	BGIBMGA002026	Gene000081	413	46323	8.62	mito	BTB	1
	BTBD4	BGIBMGA007329	Gene001228	229	25941	5.91	nucl	BTB	3
	BTBD5	BGIBMGA002966	Gene002082	450	52799	5.87	cyto	BTB	4
	BTBD6	BGIBMGA003094	Gene002316	366	40210	8.33	cyto	BTB	4
	BTBD7	BGIBMGA006426	Gene003326	412	45681	5.00	nucl	BTB	6
	BTBD8	BGIBMGA006356	Gene003489	417	48190	6.13	cyto	BTB	6
	BTBD9	BGIBMGA008687	Gene004103	288	31902	8.08	nucl	BTB	7
	BTBD10	No	Gene005182	787	88357	4.89	nucl	BTB	9
	BTBD11	BGIBMGA001715	Gene006003	517	56039	9.12	nucl	BTB	11
	BTBD12	BGIBMGA009340	Gene008379	340	39122	6.04	cyto	BTB	14
	BTBD13	BGIBMGA007642	Gene008555	361	41882	5.21	cyto	BTB	15
	BTBD14	BGIBMGA007941	Gene008559	252	29069	5.18	cyto	BTB	15
	BTBD15	No	Gene016199	128	14575	7.73	cyto	BTB	15
	BTBD16	BGIBMGA001575	Gene012166	1367	155410	4.96	nucl	BTB	21
	BTBD17	BGIBMGA011238	Gene013474	538	61092	6.06	mito	BTB	23
	BTBD18	No	Gene013871	379	42044	8.04	nucl	BTB	24
	BTBD19	BGIBMGA000033	Gene015082	287	33573	5.96	cyto	BTB	26
	BTBD20	BGIBMGA014444	Gene016272	118	13280	8.96	cyto	BTB	Unknown
	BTBD21	BGIBMGA011597	Gene016080	182	19966	5.69	nucl	BTB	Unknown

BTB-ANK	ABTB1	BGIBMGA001072	Gene008052	1133	120246	8.21	nucl	ANK-BTB	13
	ABTB2	BGIBMGA004120	Gene011234	1236	139090	8.25	nucl	ANK-BTB	19

BTB-BACK	BBTB1	BGIBMGA006106	Gene001734	593	66528	5.74	cyto	BTB-BACK	4
	BBTB2	BGIBMGA013583	Gene003242	514	59279	5.85	nucl	BTB-BACK	6
	BBTB3	BGIBMGA005281	Gene004340	359	40022	7.03	nucl	BTB-BACK	8
	BBTB4	BGIBMGA002863	No	669	75503	5.42	cyto	BTB-BACK	10
	BBTB5	BGIBMGA011825	Gene006515	405	44924	5.43	nucl	BTB-BACK	11

BTB-BACK-PHR	BBP1	BGIBMGA007155	Gene012272	505	56012	5.47	cyto	BTB-BACK-PHR	21
	BBP2	BGIBMGA005139	Gene014633	535	59037	5.44	cyto/m	BTB-BACK-PHR	25

BTB-BACK-Kelch	BBK1	BGIBMGA007372	Gene001129	612	65335	5.09	cyto	BTB-BACK-Kelch	3
	BBK2	BGIBMGA006093	Gene001685	646	71864	8.00	nucl	BTB-BACK-Kelch	4
	BBK3	BGIBMGA011859	Gene006441	628	71412	7.44	nucl	BTB-BACK-Kelch	11
	BBK4	BGIBMGA010339	Gene007321	686	77416	5.23	cyto/m	BTB-BACK-Kelch	12
	BBK5	BGIBMGA004699	Gene012782	524	58082	8.53	nucl	BTB/BACK/Kelch	22
	BBK6	BGIBMGA011506	Gene013128	611	66894	5.90	cyto	BTB/BACK/Kelch	23

BTB-Kelch	KBTB	BGIBMGA012932	Gene009636	798	90009	6.23	cyto	BTB-Kelch	16

BTB-ZF (C2H2)	ZBTB1	BGIBMGA002080	Gene000027	370	42226	6.44	nucl	BTB-C2H2 zinc finger	1
	ZBTB2	BGIBMGA002948	Gene002051	269	30496	5.41	nucl	BTB-C2H2 zinc finger	4
	ZBTB3	BGIBMGA003766	Gene002371	914	99826	8.50	nucl	BTB-C2H2 zinc finger	4
	ZBTB4	BGIBMGA002702	Gene002544	563	62824	6.79	nucl	BTB-C2H2 zinc finger	5
	ZBTB5	BGIBMGA006492	Gene003412	269	30293	6.78	nucl	BTB-C2H2 zinc finger	6
	ZBTB6	BGIBMGA009907	Gene004719	456	50728	7.74	nucl	BTB-C2H2 zinc finger	8
	ZBTB7	BGIBMGA012517	Gene005005	581	65916	5.97	nucl	BTB-C2H2 zinc finger	9
	ZBTB8	BGIBMGA006736	Gene005448	435	48049	6.02	nucl	BTB-C2H2 zinc finger	10
	ZBTB9	BGIBMGA007530	Gene009012	535	59636	6.01	nucl	BTB-C2H2 zinc finger	15
	ZBTB10	BGIBMGA010850	Gene012859	705	77364	6.55	nucl	BTB-C2H2 zinc finger	22
	ZBTB11	BGIBMGA000316	Gene012612	464	51255	6.25	nucl	BTB-C2H2 zinc finger	22
	ZBTB12	BGIBMGA000376	No	511	58344	9.08	nucl	BTB-C2H2 zinc finger	22
	ZBTB13	No	Gene015971	292	33373	9.27	nucl	BTB-C2H2 zinc finger	Unknown

BTB-ZF (FLYWCH)	ZBTB14	BGIBMGA005898	Gene007418	299	33425	8.78	cyto	BTB-FLYWCH zinc finger	12
	ZBTB15	BGIBMGA005879	Gene007419	338	38692	4.90	cyto	BTB-FLYWCH zinc finger	12
	ZBTB16	No	Gene007155	389	44521	6.32	cyto	BTB-FLYWCH zinc finger	12

BTB-HTH	HBTB1	BGIBMGA008942	Gene001524	503	56414	8.67	nucl	BTB-HTH DNA binding motif	3
	HBTB2	BGIBMGA001210	Gene007758	516	57152	8.61	nucl	BTB-HTH DNA binding motif	13

MATH-BTB	MBTB	BGIBMGA012495	Gene005056	434	48266	7.07	nucl	MATH-BTB	9

Note: cyto: cytosol; nucl: nuclear; mito: mitochondrion; m: mitochondrion.

**Table 2:** Number variation of the members of each BTB protein subfamily in the silkworm and other insects.

Subfamily	Silkworm (Lepidoptera)	Fruit fly (Diptera)	Honey bee (Hymenoptera)	Red flour beetle (Coleoptera)	Monarch butterfly (Lepidoptera)
BTB only	22	8	12	11	13
BTB-ANK	2	2	2	2	2
BTB-BACK	5	4	2	5	4
BTB-BACK-PHR	2	—	1	—	2
BTB-BACK-Kelch	6	7	7	10	6
BTB-Kelch	1	1	—	1	1
BTB-ZF (C2H2)	12	11	14	15	15
BTB-ZF (FLYWCH)	3	2	1	1	5
BTB-HTH	2	7	5	4	2
MATH-BTB	1	1	1	1	1
RhoBTB	—	1	—	1	1
Others	—	—	1	4	1

Total	56	44	46	55	53

Note: — represents no identification.

## Abbreviations

BTB: Bric-a-brac, tramtrack, and broad complex
ZF: Zinc finger
BACK: BTB and C-terminal Kelch
MATH: Meprin and TRAF homology
ANK: Ankyrin
Rho: Ras homology
BR-C: Broad complex.
