# Supplementary material for: Identification and Expression Profiling of the BTB Domain-Containing Protein Gene Family in the Silkworm, Bombyx mori
Source: Int J Genomics. 2014 May 6;2014:865065. doi: 10.1155/2014/865065 (PMC4033408; doi:10.1155/2014/865065)
Supplement: Supplementary file 1 — Table S1: List of the primers used in this study. Table S2: Inventory of BTB protein genes in other four insect species, including the fruit fly, honey bee, red flour beetle, and monarch butterfly. [file 865065.f1.pdf]

Table S1 List of primers used in this study

Gene	Sense (5' to 3')	Antisense (5' to 3')
BTBD7	AGAACATCATTCAAACGGGGAG	ATCGGATGTTGTGTTGGATTCA
BTBD8	GCTTATTGGGCAACAGCGAGT	TCTGCAAAACCTCTGCTGTACCT
BTBD13	TAGTCATGGCAGTGAAATGGAAA	GAAATGCAATGGCTATGTACAGG
BTBD19	CTGAGTCGTCGCCAAAGCAT	CGATGGGCCAAATGTATGC
ABTB2	TCACGAAGAGTCAGTCAGTTAGGA	AAGATATACGATTTGATGGCGC
BBP2	TGTCTCCACCACACACTGTATCAC	GCAGCGTTTCTCTCTCGAAC
BBK1	ACGCTGATGGCGACAGAGAA	GCATCTTGTCTCAACCTCGCTA
BBK2	CCCACACCTCGGAGAAACATC	CAGTGAACATCGCTCGGAAGT
BBK4	TGCTATGCGACGCTATTGTG	GCGGAGGTAGGCGTACTCAA
BBK6	TGATAAGGGACACGCTGAAGTAG	TGAGTCAATGCGCTCAAGTGTTA
KBTB	GGAACCCGAATGAACTGGATA	ATACCCATACAGCCCATTTTAGTC
ZBTB2	GAAGAAGATTCGGCTAAGTCGC	AGCTGTGCTTGTCTGGGGAT
ZBTB5	CGCTGGAACAACCACCCTAA	GGCCAACATTTACTTCCCCC
ZBTB6	CGACGGGAAAAGCCTCAAA	CCTGGGCAGCGTCATTGTG
ZBTB8	GGTACTTTCGGCTTGTAGTCCATA	GCTTTTGAAGAAGAGGTAGAATGC
ZBTB13	AGATGGGCATATGGTGCGAGT	TCTAATGTGAAGTGCTTTCCCAG
ZBTB14	GCACCAAGTCATAATCGCTCTG	TCTTCAATTCTTTCGCTGCCTC
MBTB	ATCATCGACTTTCTCAGCTGGTG	GTCTTTTCCTTGCACGAATCTGT
RpL3	TCGTCATCGTGGTAAGGTCAA	TTTGTATCCTTTGCCCTTGGT
