# Supplementary material for: Identification and Expression Profiling of the BTB Domain-Containing Protein Gene Family in the Silkworm, Bombyx mori
Source: Int J Genomics. 2014 May 6;2014:865065. doi: 10.1155/2014/865065 (PMC4033408; doi:10.1155/2014/865065)
Supplement: Supplementary file 2 [file 865065.f2.pdf]

Table S2_Inventory of BTB proteins in other insects

Subfamily	*Drosophila melanogaster*			*Apis mellifera*			*Tribolium castaneum*			*Danaus plexippus*		
	ID	Symbol	Domain	ID	Symbol	Domain	ID	Symbol	Domain	ID	Symbol	Domain
BTB only	CG11275	DmBTBD1	BTB	GB10603	AmBTBD1	BTB	GLEAN_00415	TcBTBD1	BTB	DPOGS200522-PA	DpBTBD1	BTB
	CG12052	DmBTBD2	BTB	GB10633	AmBTBD2	BTB	GLEAN_03621	TcBTBD2	BTB	DPOGS202476-PA	DpBTBD2	BTB
	CG13917	DmBTBD3	BTB	GB10960	AmBTBD3	BTB	GLEAN_06410	TcBTBD3	BTB	DPOGS202712-PA	DpBTBD3	BTB
	CG3726	DmBTBD4	BTB	GB12094	AmBTBD4	BTB	GLEAN_09535	TcBTBD4	BTB	DPOGS203471-PA	DpBTBD4	BTB
	CG5738	DmBTBD5	BTB	GB14408	AmBTBD5	BTB	GLEAN_10343	TcBTBD5	BTB	DPOGS204086-PA	DpBTBD5	BTB
	CG6118	DmBTBD6	BTB	GB14649	AmBTBD6	BTB	GLEAN_10446	TcBTBD6	BTB	DPOGS205406-PA	DpBTBD6	BTB
	CG8924	DmBTBD7	BTB	GB14696	AmBTBD7	BTB	GLEAN_11439	TcBTBD7	BTB	DPOGS206029-PA	DpBTBD7	BTB
	CG9343	DmBTBD8	BTB	GB15064	AmBTBD8	BTB	GLEAN_13203	TcBTBD8	BTB	DPOGS208783-PA	DpBTBD8	BTB
				GB16366	AmBTBD9	BTB	GLEAN_15126	TcBTBD9	BTB	DPOGS208924-PA	DpBTBD9	BTB
				GB18588	AmBTBD10	BTB	GLEAN_15970	TcBTBD10	BTB	DPOGS209727-PA	DpBTBD10	BTB
				GB20142	AmBTBD11	BTB	GLEAN_08426	TcBTBD11	BTB	DPOGS210632-PA	DpBTBD11	BTB
				GB30549	AmBTBD12	BTB				DPOGS212156-PA	DpBTBD12	BTB
										DPOGS213543-PA	DpBTBD13	BTB
BTB-ankyrin	CG11250	DmABTB1	ANK-BTB	GB17271	AmABTB1	Ank-BTB	GLEAN_07059	TcABTB1	ANK-BTB	DPOGS215957-PA	DpABTB1	Ank-BTB
	CG8060	DmABTB2	ANK-RCC1-BTB	GB19019	AmABTB2	BTB-ANK-FYVE	GLEAN_09632	TcABTB2	ANK-RCC1-BTB-BTB	DPOGS214313-PA	DpABTB2	Ank-Tetraspannin-BTB
BTB-BACK	CG12423	DmBBTB1	BTB-BACK	GB11719	AmBBTB1	BTB-BACK	GLEAN_00949	TcBBTB1	BTB-BACK	DPOGS201858-PA	DpBBTB1	BTB-BACK
	CG1826	DmBBTB2	BTB-BACK	GB12345	AmBBTB2	BTB-BACK	GLEAN_09541	TcBBTB2	BTB-BACK	DPOGS204938-PA	DpBBTB2	BTB-BACK
	CG1841	DmBBTB3	BTB-BACK				GLEAN_14707	TcBBTB3	BTB-BACK	DPOGS205931-PA	DpBBTB3	BTB-BACK
	CG16952	DmBBTB4	BTB-BTB-BACK				GLEAN_12366	TcBBTB4	BTB-BACK-C2H2	DPOGS212344-PA	DpBBTB4	BTB-BACK
							GLEAN_01623	TcBBTB5	BTB-BTB-BACK			
BTB-BACK-PHR				GB17866	AmBBP	BTB-BACK-PHR				DPOGS201089-PA	DpBBP1	BTB-BACK-PHR
										DPOGS214572-PA	DpBBP2	BTB-BACK-PHR
BTB-BACK-Kelch	CG17754	DmBBK1	BTB-BACK-Kelch	GB11323	AmBBK1	BTB-BACK-Kelch	GLEAN_05972	TcBBK1	BTB-BACK-Kelch	DPOGS201838-PA	DpBBK1	BTB-BACK-Kelch
	CG1812	DmBBK2	BTB-BACK-Kelch	GB12199	AmBBK2	BTB-BACK-Kelch	GLEAN_06676	TcBBK2	BTB-BACK-Kelch	DPOGS203216-PA	DpBBK2	BTB-BACK-Kelch
	CG3571	DmBBK3	BTB-BACK-Kelch	GB13707	AmBBK3	BTB-BACK-Kelch	GLEAN_10383	TcBBK3	BTB-BACK-Kelch	DPOGS206094-PA	DpBBK3	BTB-BACK-Kelch
	CG3962	DmBBK4	BTB-BACK-Kelch	GB13968	AmBBK4	BTB-BACK-Kelch	GLEAN_12393	TcBBK4	BTB-BACK-Kelch	DPOGS207551-PA	DpBBK4	BTB-BACK-Kelch
	CG6224	DmBBK5	BTB-BACK-Kelch	GB14950	AmBBK5	BTB-BACK-Kelch	GLEAN_12547	TcBBK5	BTB-BACK-Kelch	DPOGS208488-PA	DpBBK5	BTB-BACK-Kelch
	CG7210	DmBBK6	BTB-BACK-Kelch	GB14989	AmBBK6	BTB-BACK-Kelch	GLEAN_12628	TcBBK6	BTB-BACK-Kelch	DPOGS213184-PA	DpBBK6	BTB-BACK-Kelch
	CG9426	DmBBK7	BTB-BACK-Kelch	GB19993	AmBBK7	BTB-BACK-Kelch	GLEAN_13883	TcBBK7	BTB-BACK-Kelch			
							GLEAN_15037	TcBBK8	BTB-BACK-Kelch			
							GLEAN_16270	TcBBK9	BTB-BACK-Kelch			
							GLEAN_00194	TcBBK10	G_patch-BTB-BACK-Kelch			
BTB-Kelch	CG3711	DmKBTB	BTB-Kelch				GLEAN_09594	TcKBTB	BTB-Kelch	DPOGS207008-PA	DpKBTB	Kelch-BTB
BTB-ZF(C2H2)	CG11491	DmZBTB1	BTB-C2H2	GB10751	AmZBTB1	BTB-C2H2	GLEAN_01442	TcZBTB1	BTB-C2H2	DPOGS201452-PA	DpZBTB1	BTB-C2H2
	CG12236	DmZBTB2	BTB-C2H2	GB11420	AmZBTB2	BTB-C2H2	GLEAN_03097	TcZBTB2	BTB-C2H2	DPOGS202607-PA	DpZBTB2	BTB-C2H2
	CG1856	DmZBTB3	BTB-C2H2	GB12001	AmZBTB3	BTB-C2H2	GLEAN_05474	TcZBTB3	BTB-C2H2	DPOGS203320-PA	DpZBTB3	BTB-C2H2
	CG31666	DmZBTB4	BTB-C2H2	GB13949	AmZBTB4	BTB-C2H2	GLEAN_05851	TcZBTB4	BTB-C2H2	DPOGS204256-PA	DpZBTB4	BTB-C2H2
	CG32121	DmZBTB5	BTB-C2H2	GB14319	AmZBTB5	BTB-C2H2	GLEAN_05966	TcZBTB5	BTB-C2H2	DPOGS205830-PA	DpZBTB5	BTB-C2H2
	CG32611	DmZBTB6	BTB-C2H2	GB15346	AmZBTB6	BTB-C2H2	GLEAN_10050	TcZBTB6	BTB-C2H2	DPOGS207435-PA	DpZBTB6	BTB-C2H2
	CG4807	DmZBTB7	BTB-C2H2	GB17083	AmZBTB7	BTB-C2H2	GLEAN_11137	TcZBTB7	BTB-C2H2	DPOGS207933-PA	DpZBTB7	BTB-C2H2
	CG5575	DmZBTB8	BTB-C2H2	GB18560	AmZBTB8	BTB-C2H2	GLEAN_12894	TcZBTB8	BTB-C2H2	DPOGS207965-PA	DpZBTB8	BTB-C2H2
	CG6384	DmZBTB9	BTB-C2H2	GB18625	AmZBTB9	BTB-C2H2	GLEAN_12994	TcZBTB9	BTB-C2H2	DPOGS209522-PA	DpZBTB9	BTB-C2H2

	CG6765	DmZBTB10	BTB-C2H2	GB19568	AmZBTB10	BTB-C2H2	GLEAN_13098	TcZBTB10	BTB-C2H2	DPOGS210888-PA	DpZBTB10	BTB-C2H2
	CG14307	DmZBTB11	BTB-C2H2	GB30150	AmZBTB11	BTB-C2H2	GLEAN_13787	TcZBTB11	BTB-C2H2	DPOGS210911-PA	DpZBTB11	BTB-C2H2
				GB30158	AmZBTB12	BTB-C2H2	GLEAN_13792	TcZBTB12	BTB-C2H2	DPOGS211010-PA	DpZBTB12	BTB-C2H2
				GB17617	AmZBTB13	BTB-C2H2	GLEAN_13842	TcZBTB13	BTB-C2H2	DPOGS211707-PA	DpZBTB13	BTB-C2H2
				GB30349	AmZBTB14	DUF3591-BROMO-BTB-C2H	GLEAN_00589	TcZBTB14	BTB-C2H2_TTF-C2H2	DPOGS212369-PA	DpZBTB14	BTB-C2H2
							GLEAN_12869	TcZBTB15	BTB-C2H2-BEN	DPOGS214291-PA	DpZBTB15	BTB-C2H2
	CG31160	DmZBTB12	BTB-FLYWCH	GB11400	AmZBTB15	FLYWCH-C2H2_BED-BTB	GLEAN_16226	TcZBTB16	BTB-FLYWCH	DPOGS205112-PA	DpZBTB16	BTB-FLYWCH
	CG32491	DmZBTB13	BTB-FLYWCH							DPOGS205804-PA	DpZBTB17	BTB-FLYWCH
BTB-ZF(FLYWCH)										DPOGS205805-PA	DpZBTB18	BTB-FLYWCH
										DPOGS207614-PA	DpZBTB19	BTB-FLYWCH
										DPOGS208236-PA	DpZBTB20	BTB-FLYWCH
				GB14194	AmHBTB1	BTB-HTH_psq	GLEAN_03349	TcHBTB1	BTB-HTH_psq	DPOGS203925-PA	DpHBTB1	BTB-HTH_psq
	CG11494	DmHBTB1	BTB-HTH_psq	GB14243	AmHBTB2	BTB-HTH_psq	GLEAN_06481	TcHBTB2	BTB-HTH_psq	DPOGS215980-PA	DpHBTB2	BTB-HTH_psq
	CG15812	DmHBTB2	BTB-HTH_psq	GB15091	AmHBTB3	BTB-HTH_psq	GLEAN_07162	TcHBTB3	BTB-HTH_psq			
BTB-HTH	CG16778	DmHBTB3	BTB-HTH_psq	GB16756	AmHBTB4	BTB-HTH_psq	GLEAN_03616	TcHBTB4	BTB-HTH_psq			
	CG2368	DmHBTB4	BTB-HTH_psq	GB17640	AmHBTB5	BTB-HTH_psq						
	CG7230	DmHBTB5	BTB-HTH_psq									
	CG9097	DmHBTB6	BTB-HTH_psq									
	CG9102	DmHBTB7	BTB-HTH_psq									
MATH-BTB	CG9924	DmMBTB	MATH-BTB	GB17428	AmMBTB	MATH-BTB	GLEAN_00632	TcMBTB	MATH-BTB	DPOGS201231-PA	DpMBTB	MATH-BTB
RhoBTB	CG5701	DmRhoBTB	RhoBTB-BTB				GLEAN_12457	TcRhoBTB	RhoBTB-BTB	DPOGS210262-PA	DpRhoBTB	RhoBTB-BTB
				GB15293	AmDBTB	BTB-DnaJ	GLEAN_07345	TcHMBTB	BTB-HMG	DPOGS206600-PA	DpLRBTB	LIM-Rap_GAP-BTB
Others							GLEAN_01280	TcRBTB	BTB-RRM			
							GLEAN_11466	TcGBTB	GRAM-BTB			
							GLEAN_12517	TcRNBTB	RING-NHL-BTB			
